# Comment on ‘Asian Reference Values for Handgrip Strength, Gait Speed, Five‐Times‐Sit‐to‐Stand Test, Muscle Mass and Calf Circumference’ by Grgic et al.—The Authors' Reply

**DOI:** 10.1002/jcsm.70327

**Published:** 2026-06-25

**Authors:** Jozo Grgic, Siew Ling Tey, Dieu Thi Thu Huynh, Yen Ling Low, Zeljko Pedisic, Nina Schaller, Vanessa Kristina Wazny, Weilan Wang, Yasuhiko Saito, Ravindra P. Rannan‐Eliya, Hala Ghattas, Monique Chaaya, Carlos Mendes de Leon, Preeti Gupta, Ecosse L. Lamoureux, Mythily Subramaniam, Edimansyah Abdin, Rahul Malhotra, Angelique Chan, Bayasgalan Tumenbayar, Oyunbileg Luvsandavaajav, Bolormaa Enkhtuvshin, Norma Mansor, Halimah Awang, Andrea B. Maier

**Affiliations:** ^1^ NUS Academy for Healthy Longevity, Yong Loo Lin School of Medicine National University of Singapore Singapore Singapore; ^2^ Department of Sports Science and Physical Education The Chinese University of Hong Kong Hong Kong China; ^3^ Abbott Nutrition Research and Development, Asia‐Pacific Centre Singapore Singapore; ^4^ School of Public Health, Li Ka Shing Faculty of Medicine The University of Hong Kong Hong Kong China; ^5^ Department for Preventive Sports Medicine and Sports Cardiology, TUM School of Medicine and Health TUM University Hospital, Technical University of Munich (TUM) Munich Germany; ^6^ College of Economics Nihon University Chiyoda Japan; ^7^ Economic Research Institute for ASEAN and East Asia Jakarta Indonesia; ^8^ Institute for Health Policy Colombo Sri Lanka; ^9^ Department of Health Promotion, Education, and Behavior, Arnold School of Public Health University of South Carolina Columbia South Carolina USA; ^10^ Department of Epidemiology and Population Health, Faculty of Health Sciences American University of Beirut Beirut Lebanon; ^11^ Department of Global Health Georgetown University School of Health Washington DC USA; ^12^ Singapore Eye Research Institute Singapore National Eye Centre Singapore Singapore; ^13^ Duke‐NUS Medical School Singapore Singapore; ^14^ Department of Ophthalmology The University of Melbourne Melbourne Victoria Australia; ^15^ Research Division Institute of Mental Health Singapore Singapore; ^16^ Saw Swee Hock School of Public Health National University Singapore Singapore Singapore; ^17^ Centre for Ageing Research & Education (CARE) Duke‐NUS Medical School Singapore Singapore; ^18^ Programme in Health Services and Systems Research (HSSR) Duke‐NUS Medical School Singapore Singapore; ^19^ Postgraduate Training Institute Mongolian National University of Medical Sciences Ulaanbaatar Mongolia; ^20^ Graduate School of Public Health Inje University Gimhae Republic of Korea; ^21^ Intermed Hospital Ulaanbaatar Mongolia; ^22^ Social Wellbeing Research Centre Universiti Malaya Kuala Lumpur Malaysia; ^23^ Healthy Longevity Translational Research Program, Yong Loo Lin School of Medicine National University of Singapore Singapore; ^24^ Department of Human Movement Sciences, @AgeAmsterdam, Faculty of Behavioural and Movement Sciences Vrije Universiteit Amsterdam, Amsterdam Movement Sciences Amsterdam the Netherlands

We thank Lim et al. [1] for their interest in our work [2]. In their letter [1], Lim and colleagues made a comparison between reference values in our study [2] and the cut‐offs proposed in their 2025 Asian Working Group for Sarcopenia (AWGS) update [3]. The AWGS 2025 cut‐offs (co‐authored, among others, by all authors of the Lim et al. letter [1]) were based on expert opinion/consensus and reference values from a pooled analysis of data from Taiwan, Japan and Malaysia [4] (co‐authored, among others, by three authors of the Lim et al. letter [1]). Our analyses [2] included national‐representative samples from China, India, Indonesia, Israel, Japan, Lebanon, Malaysia, Mongolia, Philippines, Republic of Korea, Singapore and Sri Lanka. Given that normative values for muscle strength vary between countries and regions, findings of our study [2] and previous studies [3, 4] may not be directly comparable.

Lim et al. [1] expressed concern about the heterogeneity of pooled samples across different measures of muscle health in our study. Our analysis was based on the best available evidence at the time of the study, including 18 cohorts for handgrip strength (*n* = 277 921); five cohorts for 4‐m gait speed test (*n* = 139 121); four cohorts for Five‐Times‐Sit‐to‐Stand Test (FTSST; *n* = 55 235), skeletal muscle mass (*n* = 49 309) and 2.5‐m gait speed test (*n* = 13 474); and two cohorts for calf circumference (*n* = 19 465). The previous pooled analysis [4] included seven cohorts for handgrip strength (*n* = 16 730); five cohorts for calf circumference (*n* = 13 209) and various gait speed tests (*n* = 13 421); three cohorts for FTSST (*n* = 7785) and appendicular muscle mass measured by dual‐energy X‐ray absorptiometry (DXA; *n* = 11 989); and two cohorts for appendicular muscle mass measured by bioelectrical impedance analysis (BIA; *n* = 4872). Despite the heterogeneity of pooled samples across different outcomes and limited representation of the overall Asian population, the reference values from the previous pooled analysis [4] proved to be useful for deriving the AWGS 2025 cut‐offs [3]. Given that our findings [2] were based on a substantially larger and more diverse dataset with a broader geographic representation of Asia, they should also be considered in future updates of AWGS cut‐offs.

Given that the AWGS 2025 cut‐offs generally correspond to mid‐ to upper‐range percentiles in our study, Lim et al. [1] claimed that this ‘strongly suggests’ a discrepancy of our findings from true normative values. Such a statement is not justified, because this difference between findings from two studies could equally so suggest a discrepancy of their findings [4] from true normative values. Moreover, our stratified analyses showed higher values for handgrip strength, 2.5‐m gait speed and skeletal muscle mass in East Asia compared to pan‐Asian values, or those from Southeast or South Asia [2]. Given that the data in the previous pooled analysis [4] were mainly from East Asian cohorts, the differences from our findings are in the expected and logical direction [2, 4].

In their letter [1], Lim and colleagues compared reference values for gait speed from our study with the AWGS 2025 cut‐offs [4]. However, making such a comparison would be unjustified because the AWGS 2025 update [3] provided cut‐offs for the 6‐m gait speed test, whereas reference values in our study [2] are for the 2.5‐ and 4‐m gait speed tests. Gait speed tests using different distances may not be directly comparable [5].

Lim et al. [1] also highlighted the limitation of combining data from different cohorts with different measurement protocols. We had already acknowledged and discussed this common limitation of pooled analyses of large‐scale, multi‐national cohorts in our paper [2]. It is worth noting that similar considerations also apply to the previous pooled analysis [4], as it combined data across different cohorts with different measurement protocols. Despite this limitation, the findings of the previous analysis [4] proved to be useful when developing the AWGS 2025 cut‐offs [3].

When referring to our analysis for handgrip strength, Lim et al. [1] stated that ‘it was unclear if the average or maximum value was used’. This is not correct, because in our paper [2], we specifically wrote: ‘Reference values for handgrip strength were calculated for the dominant hand, following the guidelines from the Asian Working Group for Sarcopenia’—which recommends using the maximum value [6].

Additionally, Lim et al. [1] raised concerns regarding the combination of DXA‐ and BIA‐derived measurements. We had already explicitly acknowledged this limitation in our paper [2]:

Additionally, there are limitations in combining data from BIA and DXA, given that these methods provide somewhat different measures. Hence, while our pan‐Asian reference values for skeletal muscle mass may serve as a general guide, using the country‐ and device‐specific reference values may enable a more accurate interpretation.

Lim et al. [1] also stated that differences in gait speed testing protocols (2.5‐ and 4‐m) may have influenced our results. This statement is not applicable to our study [2], as we conducted separate analyses for 2.5‐m test and 4‐m test. Our approach differed from their previous pooled analysis [4], which combined data from the 4‐, 5‐, 6‐ and 10‐m gait speed tests into a single, pooled analysis.

Lim et al. [1] further suggested that differences in cohort focus may limit comparability of results. As specified in our inclusion criteria [2], the analyses were restricted to national cohort studies with community‐dwelling samples from the general population. In comparison, the previous pooled analysis [4] combined data from subnational, geographically confined, single‐city, district‐based and ageing‐focused research cohorts with differing sampling frames, and despite this limitation, it was used to inform the AWGS 2025 cut‐offs [3].

When referring to the fact that we combined data collected from 2004 to 2024, Lim et al. [1] reiterated a point on secular trends that we had made in the limitations section of our paper [2]. Again, the same limitation also applies to the previous pooled analysis [4], which combined data from an even longer period (from 1999 to 2022).

In summary, our study on reference values represents the largest pooled analysis of individual participant data from different national cohorts across Asia [2]. Notwithstanding the limitations that we had discussed in detail in our paper, it provides the largest available set of age‐ and sex‐specific reference values for several indicators of muscle health across Asian national population‐based cohorts [2]. As such, the reference values presented in our study [2] merit consideration in future refinements of criteria developed by various working groups on sarcopenia.

## Conflicts of Interest

S. L. T., D. T. T. H. and Y. L. L. are employees of Abbott Nutrition (funder of the original study) but did not have access to individual data and were not involved in the data analysis. The other authors declare no conflicts of interest.

## Data Availability

Data sharing is not applicable to this article as no datasets were generated or analysed during the current study.

## References

[1] W.‐S. Lim , P. Assantachai , R. Merchant , et al., “Comment on “Asian Reference Values for Handgrip Strength, Gait Speed, Five‐Times‐Sit‐to‐Stand Test, Muscle Mass and Calf Circumference” by Grgic et al.,” Journal of Cachexia, Sarcopenia and Muscle 17, no. 3 (2026): e70317, 10.1002/jcsm.70317. 42306827 PMC13273395

[2] J. Grgic , S. L. Tey , D. T. T. Huynh , et al., “Asian Reference Values for Handgrip Strength, Gait Speed, Five‐Times‐Sit‐to‐Stand Test, Muscle Mass and Calf Circumference,” Journal of Cachexia, Sarcopenia and Muscle 17, no. 1 (2026): e70216, 10.1002/jcsm.70216. 41674016 PMC12894420

[3] L. K. Chen , F. Y. Hsiao , M. Akishita , et al., “A Focus Shift From Sarcopenia to Muscle Health in the Asian Working Group for Sarcopenia 2025 Consensus Update,” Nature Aging 5, no. 11 (2025): 2164–2175. 41188603 10.1038/s43587-025-01004-y

[4] L. K. Chen , L. C. Meng , L. N. Peng , et al., “Mapping Normative Muscle Health Metrics Across the Aging Continuum: A Multinational Study Pooling Data From Eight Cohorts in Japan, Malaysia and Taiwan,” Journal of Cachexia, Sarcopenia and Muscle 16 (2025): e13731. 39971708 10.1002/jcsm.13731 PMC11839280

[5] A. K. Stuck , M. Bachmann , P. Füllemann , et al., “Effect of Testing Procedures on Gait Speed Measurement: A Systematic Review,” PLoS ONE 15 (2020): e0234200. 32479543 10.1371/journal.pone.0234200 PMC7263604

[6] L. K. Chen , J. Woo , P. Assantachai , et al., “Asian Working Group for Sarcopenia: 2019 Consensus Update on Sarcopenia Diagnosis and Treatment,” Journal of the American Medical Directors Association 21 (2020): 300–307.e2. 32033882 10.1016/j.jamda.2019.12.012

